# Occurrence rates and risk factors of in-hospital venous thromboembolism, major bleeding, and death in patients receiving fondaparinux after orthopedic surgery or trauma surgery

**DOI:** 10.1007/s11845-023-03289-7

**Published:** 2023-02-14

**Authors:** Ya Ding, Bowen Han, Bin Yuan, Mingjun Nie, Renyang Liu, Ming Zhao, Hongliang Wang

**Affiliations:** 1 Department of Orthopedics, Anhui Spinal Deformities Clinical Medical Research Center, Fuyang People’s Hospital, No. 501 Sanqing Road, Fuyang, Anhui 236000 China; 2 Department of Orthopedics, The First Affiliated Hospital of Xinxiang Medical University, Xinxiang, 453000 China; 3 Department of Orthopedics, Xinyi People’s Hospital, Xuzhou, 221400 China; 4 Department of Orthopedics, Affiliated Hospital of Jiangsu University, Zhenjiang, 212001 China; 5 Department of Orthopedics, Xiangyang Central Hospital, Affiliated Hospital of Hubei University of Arts and Science, Xiangyang, 441021 China; 6 Department of Traumatology, The Affiliated Taian City Central Hospital of Qingdao University, Taian, 271000 China

**Keywords:** Adverse events, Fondaparinux, Major bleeding, Orthopedic surgery or trauma surgery, Venous thromboembolism

## Abstract

**Aim:**

Fondaparinux is a synthetic anticoagulant that inhibits thrombosis by suppressing factor Xa. The efficacy of fondaparinux for orthopedic surgeries has been revealed by several foreign studies; however, relevant evidence in Chinese patients is lacking. This study intended to investigate the occurrence rate and risk factors of in-hospital venous thromboembolism (VTE), major bleeding, and death in patients receiving fondaparinux after orthopedic surgery or trauma surgery.

**Methods:**

Totally, 1258 patients who received fondaparinux after orthopedic surgery or trauma surgery were retrospectively enrolled. Meanwhile, in-hospital VTE, major bleeding, and death were obtained for assessment. Besides, adverse events were recorded.

**Results:**

The occurrence rates of in-hospital VTE, major bleeding, and death were 2.5%, 21.8%, and 0.0%, respectively. The multivariate logistic regression analysis revealed that only age (> 60 years vs. ≤ 60 years) (odd ratios (OR) = 3.380, *P* = 0.013) was independently correlated with increased risk of in-hospital VTE. Additionally, osteoarthritis diagnosis (OR = 3.826, *P* < 0.001), femoral head necrosis diagnosis (OR = 1.809, *P* = 0.034), hip replacement (vs. internal fracture fixation) (OR = 2.199, *P* = 0.007), knee replacement (vs. internal fracture fixation) (OR = 2.781, *P* = 0.002), and serum creatinine (abnormal vs. normal) (OR = 1.677, *P* = 0.012) were independently linked to a higher risk of in-hospital major bleeding. Moreover, the common adverse events included pain (56.6%), wound bleeding (23.0%), increased drainage (5.2%), etc.

**Conclusion:**

Fondaparinux realizes low occurrence rates of in-hospital VTE and major bleeding with tolerable adverse events in patients receiving orthopedic surgery or trauma surgery.

**Supplementary Information:**

The online version contains supplementary material available at 10.1007/s11845-023-03289-7.

## Introduction

Orthopedic surgery or trauma surgery is a surgical branch that focuses on the conditions involved in the musculoskeletal system (such as bones, joints, tendons, and ligaments) [1]. Some of the commonly performed orthopedic surgeries or trauma surgeries include hip replacement, knee replacement, meniscus repair, and internal fracture fixation [2–4]. Notably, patients undergoing orthopedic surgery or trauma surgery are often at high risk of venous thromboembolism (VTE), which is a major cause of postoperative disability and death [5–7]; in this case, anticoagulants (such as unfractionated heparin, enoxaparin, and dabigatran) are required to relieve this situation [8]. However, major bleeding is very likely to occur after receiving the anticoagulants, which also seriously affects the patient’s postoperative recovery and even carries the risk of death [9–11]. Therefore, it is crucial to investigate feasible and safe anticoagulants, which balance the occurrence of VTE and major bleeding for patients after receiving orthopedic surgery or trauma surgery.

Fondaparinux specifically binds to the activation site of antithrombin (AT) to accelerate factor Xa complex formation approximately by 300 folds, leading to rapid inhibition of factor Xa, which in turn reduces thrombin production; meanwhile, at a certain concentration, fondaparinux also forms a stable complex with platelet factor 4 (PF4) to enhance palate aggregation and activation [12, 13]. Clinically, fondaparinux is applied to prevent VTE in patients undergoing orthopedic surgery or trauma surgery [14–17]. For instance, a previous study claims that fondaparinux is effective in preventing VTE with an occurrence rate of 1.0% in patients receiving major orthopedic surgery [15]. Meanwhile, a double-arm study reveals that the risk of VTE is 6% in patients receiving fondaparinux and 8% in patients receiving enoxaparin after hip replacement surgery [16]. Moreover, fondaparinux (8.3%) obviously reduces the incidence of VTE compared with enoxaparin (19.1%) in patients receiving hip-fracture surgery [18]. However, the above-mentioned studies are carried out in countries other than China [15, 16, 18]. In addition, fondaparinux has just been launched in China recently; therefore, relevant evidence regarding fondaparinux in Chinese patients receiving orthopedic surgery or trauma surgery is scarce.

Accordingly, this multi-center, single-arm, retrospective study intended to explore the occurrence rates of in-hospital VTE, major bleeding, and death as well as their risk factors in Chinese patients receiving fondaparinux after orthopedic surgery or trauma surgery.

## Materials and methods

### Patients

This was a multi-center, single-arm, retrospective study, which reviewed 1258 patients who received fondaparinux after orthopedic surgery or trauma surgery from March 2020 to October 2022. The screened criteria contained the following: (i) aged over 18 years old, (ii) underwent orthopedic surgery or trauma surgery, and (ii) received fondaparinux for anticoagulation prophylaxis. The exclusion criteria were as follows: (i) diagnosis of deep venous thrombosis at admission (except for intramuscular venous thrombosis); (ii) had a history of anticoagulant drugs before inclusion; (iii) underwent total hip or total knee replacement over 2 times; (iv) underwent orthopedic surgery, trauma surgery, spinal surgery, ophthalmic surgery, or brain surgery within 3 months prior to admission; (v) had a history of active bleeding, gastrointestinal ulcer, vasoproliferative gastrointestinal disease, hemorrhagic stroke, or congenital or acquired bleeding disorder; (vi) had a history of acute bacterial endocarditis; (vii) had a cardiac failure of stages II–III; and (viii) had severe renal impairment (creatinine clearance rate ≤ 20 mL/min). This study had been approved by the Ethics Committee. Written informed consent was obtained from each patient.

### Medication

The administration of fondaparinux was started at 6–24 h after surgery at the dose of 2.5 mg/day by subcutaneous injection [19]. For patients with a Caprini score ≥ 2 and elective surgery, fondaparinux was administered at admission and discontinued at 24 h before surgery. Antiplatelet drugs such as aspirin and indobufen could be added for appropriate patients according to the disease status and medical advice.

### Data collection

Clinical characteristics of patients were gained, which included age, gender, history of drink, history of smoke, diagnosis, Caprini score, surgery type, medication, serum creatinine, creatinine clearance rate, and platelet count. Besides, venous thromboembolism (VTE), major bleeding, and death in hospital were obtained for assessment. VTE contained symptomatic pulmonary embolism (PE), symptomatic deep vein thrombosis (DVT), asymptomatic DVT, distal DVT, and proximal DVT. Major bleeding was assessed per the International Society on Thrombosis and Haemostasis (ISTH) guideline, which contained fatal bleeding, significant bleeding (hemoglobin declined ≥ 2 g/dL, or leading to the transfusion of red blood cells in whole blood more than two units), and bleeding at critical position (retroperitoneal, intracranial, spinal, intraocular, joint space, pericardial, compartment syndrome, etc.) [20]. Additionally, adverse events were also recorded.

### Statistics

SPSS v26.0 (IBM Corp., America) was utilized for analysis. GraphPad Prism v9.0 (GraphPad Software Inc., America) was utilized for figure plotting. Comparison analysis was performed using chi-square test or Fisher’s exact test. Factors related to VTE and major bleeding in hospital were screened using univariate and forward stepwise multivariate logistic regression analyses. *P* < 0.05 was considered significant.

## Results

### Clinical properties

The included patients who received fondaparinux after orthopedic surgery or trauma surgery had a mean age of 63.6 ± 13.7 years with 740 (58.8%) females and 518 (41.2%) males. Meanwhile, the median (interquartile range (IQR)) of the Caprini score was 10.0 (9.0–11.0). In terms of surgery type, 336 (26.7%) patients received internal fracture fixation, 352 (28.0%) patients received hip replacement, 430 (34.2%) patients received knee replacement, and 140 (11.1%) received other surgeries. In addition, 78 (6.2%) patients received antiplatelet drugs, including 33 (2.6%) patients who applied aspirin, 41 (3.3%) who administered indobufen, and 4 (0.3%) patients who used other antiplatelet drugs. Moreover, the median (IQR) values of serum creatinine, creatinine clearance rate, and platelet count were 59.6 (51.0–71.0) μmol/L, 92.3 (72.3–116.9) mL/min, and 209.0 (168.0–259.0) 10^9^/L, respectively. The specific information is listed in Table 1.

**Table 1:** Clinical characteristics

Items	Patients (*N* = 1258)
Age (years), mean ± SD	63.6 ± 13.7
Gender, no. (%)	
Female	740 (58.8)
Male	518 (41.2)
History of drink, no. (%)	117 (9.3)
History of smoke, no. (%)	129 (10.3)
Diagnosis, no. (%)	
Osteoarthritis	479 (38.1)
Fracture of hip	292 (23.2)
Femoral head necrosis	132 (10.5)
Fracture around knee joint	118 (9.4)
Fracture of distal knee joint	105 (8.3)
Fracture of femoral	75 (6.0)
Others	210 (16.7)
Caprini score, median (IQR)	10.0 (9.0–11.0)
Surgery type, no. (%)	
Internal fracture fixation	336 (26.7)
Hip replacement	352 (28.0)
Knee replacement	430 (34.2)
Others	140 (11.1)
Antiplatelet drugs, no. (%)	78 (6.2)
Aspirin	33 (2.6)
Indobufen	41 (3.3)
Others	4 (0.3)
Serum creatinine (μmol/L), median (IQR)	59.6 (51.0–71.0)
Creatinine clearance rate (mL/min), median (IQR)	92.3 (72.3–116.9)
Platelet count (10^9^/L), median (IQR)	209.0 (168.0–259.0)

*SD* standard deviation, *IQR* interquartile range

### Occurrence rates of in-hospital VTE, major bleeding, and death

**Fig. 1:**
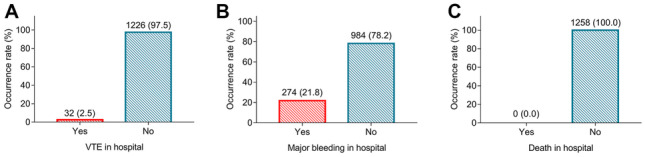
Incidence of in-hospital VTE, major bleeding, and death. The occurrence rate of in-hospital VTE (**A**), major bleeding (**B**), and death (**C**) in patients receiving fondaparinux after orthopedic surgery or trauma surgery

The occurrence rate of in-hospital VTE was 2.5% (Fig. 1A); meanwhile, the occurrence rate of in-hospital major bleeding was 21.8% (Fig. 1B); notably, no (0.0%) in-hospital death (Fig. 1C) occurred in patients receiving fondaparinux after orthopedic surgery or trauma surgery.

### Independent factors for in-hospital VTE

**Fig. 2:**
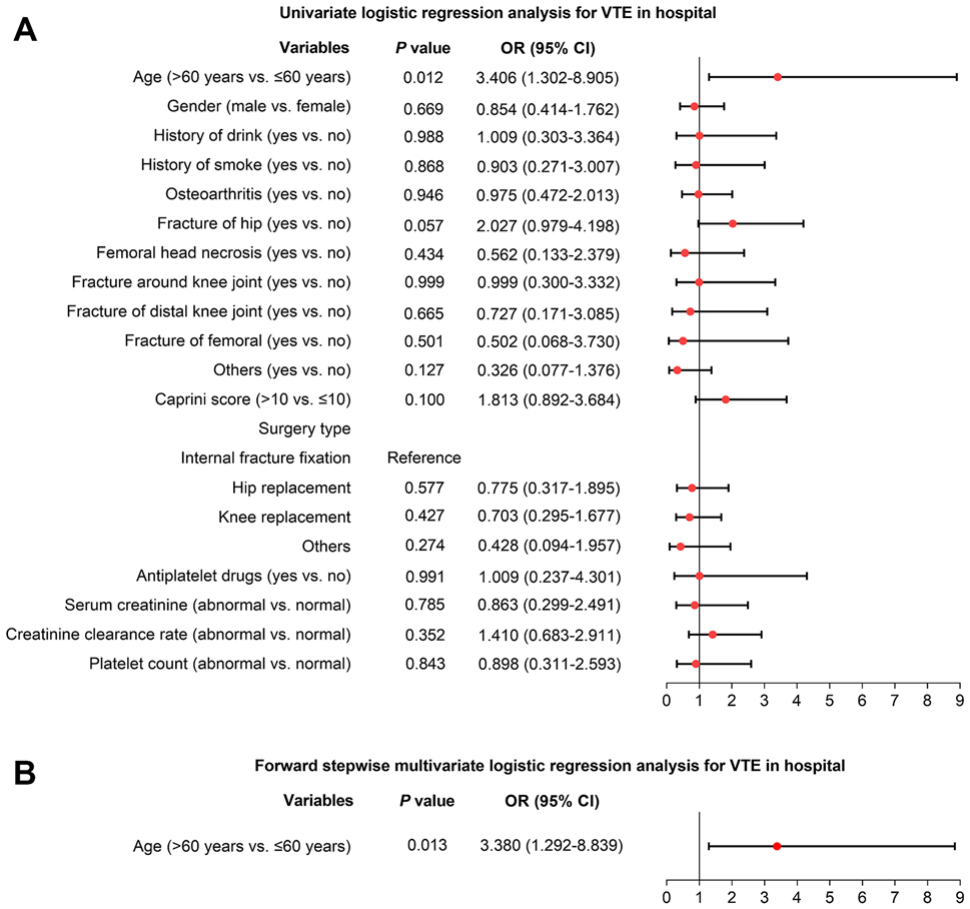
Logistic regression analysis for in-hospital VTE. Univariate logistic regression analysis for in-hospital VTE (**A**); forward stepwise multivariate logistic regression analysis for in-hospital VTE (**B**) in patients receiving fondaparinux after orthopedic surgery or trauma surgery

According to univariate logistic regression analysis, age (> 60 years vs. ≤ 60 years) (odds ratio (OR) = 3.406, *P* = 0.012) was related to higher risk of in-hospital VTE, whereas gender, history of drinking, history of smoking, diagnosis, surgery type, etc. (all *P* > 0.05), were not associated with the occurrence of in-hospital VTE in patients receiving fondaparinux after orthopedic surgery or trauma surgery (Fig. 2A).

Further forward stepwise multivariate logistic regression analysis suggested that only age (> 60 years vs. ≤ 60 years) (OR = 3.380, *P* = 0.013) was independently correlated with increased risk of in-hospital VTE in patients receiving fondaparinux after orthopedic surgery or trauma surgery (Fig. 2B).

### Independent factors for in-hospital major bleeding

**Fig. 3:**
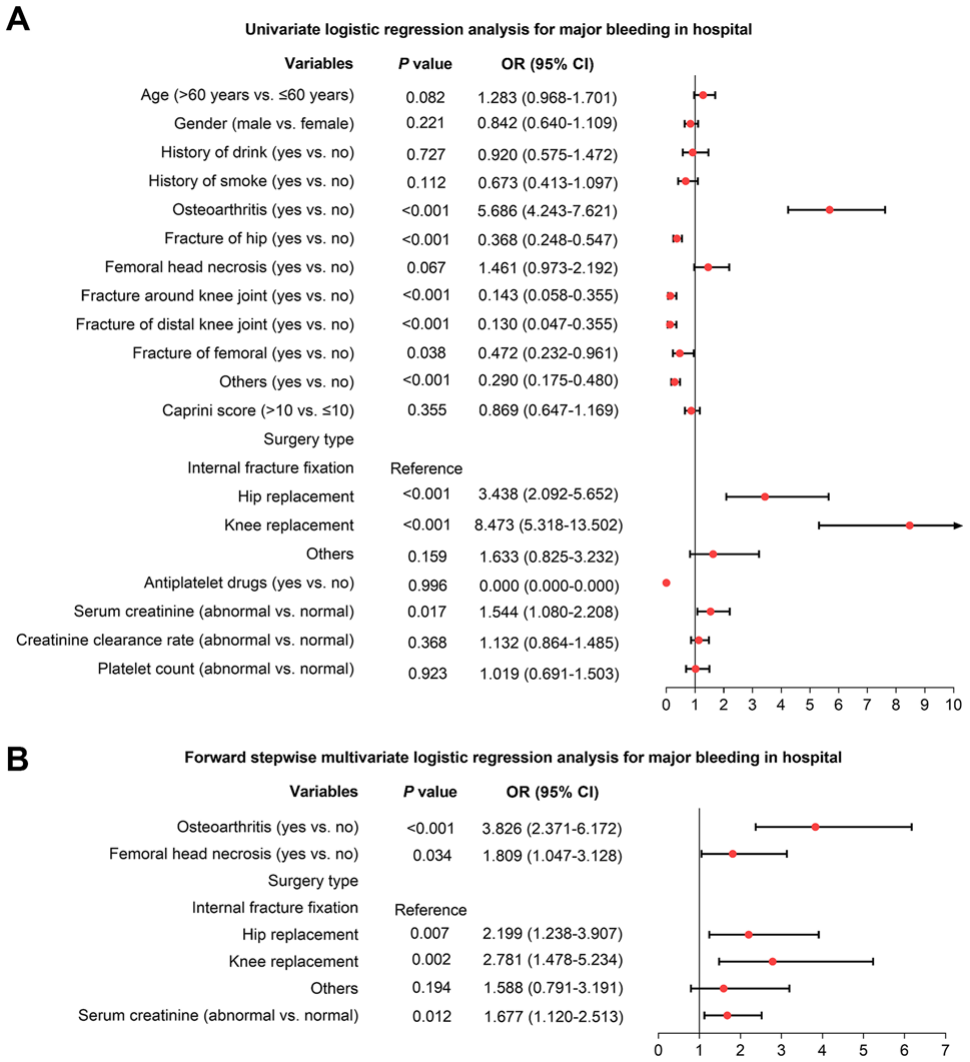
Logistic regression analysis for in-hospital major bleeding. Univariate logistic regression analysis for in-hospital major bleeding (**A**); forward stepwise multivariate logistic regression analysis for in-hospital major bleeding (**B**) in patients receiving fondaparinux after orthopedic surgery or trauma surgery

Univariate logistic regression analysis revealed that osteoarthritis (yes vs. no) (OR = 5.686, *P* < 0.001), hip replacement (vs. internal fracture fixation) (OR = 3.438, *P* < 0.001), knee replacement (vs. internal fracture fixation) (OR = 8.473, *P* < 0.001), and serum creatinine (abnormal vs. normal) (OR = 1.544, *P* = 0.017) were correlated with increased risk of in-hospital major bleeding, while fracture of hip (yes vs. no) (OR = 0.368, *P* < 0.001), fracture around knee joint (yes vs. no) (OR = 0.143, *P* < 0.001), fracture of distal knee joint (yes vs. no) (OR = 0.130, *P* < 0.001), fracture of femoral (yes vs. no) (OR = 0.472, *P* = 0.038), and other antiplatelet drugs (yes vs. no) (OR = 0.290, *P* < 0.001) were related to decreased risk of in-hospital major bleeding in patients receiving fondaparinux after orthopedic surgery or trauma surgery (Fig. 3A).

Further forward stepwise multivariate logistic regression analysis disclosed that osteoarthritis (yes vs. no) (OR = 3.826, *P* < 0.001), femoral head necrosis (OR = 1.809, *P* = 0.034), hip replacement (vs. internal fracture fixation) (OR = 2.199, *P* = 0.007), knee replacement (vs. internal fracture fixation) (OR = 2.781, *P* = 0.002), and serum creatinine (abnormal vs. normal) (OR = 1.677, *P* = 0.012) were independently linked with a higher risk of in-hospital major bleeding in patients receiving fondaparinux after orthopedic surgery or trauma surgery (Fig. 3B).

### Adverse events

In terms of adverse events, 750 (59.6%) patients suffered from surgical complications, including 712 (56.6%) patients with pain, 289 (23.0%) patients with wound bleeding, 66 (5.2%) patients with increased drainage, 15 (1.2%) patients with infection, and 245 (19.5%) patients with others. Meanwhile, 5 (0.4%) patients had adverse reactions at the injection site, and 138 (11.0%) patients had other adverse events (Table 2).

**Table 2:** Adverse events

Adverse events	Patients (*N* = 1258)
Surgical complications, no. (%)	750 (59.6)
Pain	712 (56.6)
Wound bleeding	289 (23.0)
Increased drainage	66 (5.2)
Infection	15 (1.2)
Others	245 (19.5)
Adverse reactions at injection site, no. (%)	5 (0.4)
Other adverse events, no. (%)	138 (11.0)

### Subgroup analysis of in-hospital VTE and major bleeding comparison based on different surgeries

The occurrence rates of in-hospital VTE were not different among patients receiving internal fracture fixation, hip replacement, knee replacement, and other surgeries (*P* = 0.681). In addition, the occurrence rate of in-hospital major bleeding was highest in patients receiving knee replacement, followed by patients receiving hip replacement and internal fracture fixation, and lowest in patients receiving other surgeries (*P* < 0.001) (Supplementary Fig. 1).

### Subgroup analysis of in-hospital VTE comparison based on different diagnoses

In-hospital VTE rates were not different between patients diagnosed with osteoarthritis and those who did not (*P* = 0.949), between patients diagnosed with fracture of hip and those who did not (*P* = 0.052), between patients diagnosed with femoral head necrosis and those who did not (*P* = 0.569), between patients diagnosed with fracture around knee joint and those who did not (*P* = 1.000), between patients diagnosed with fracture of distal knee joint and those who did not (*P* = 1.000), between patients diagnosed with fracture of femoral and those who did not (*P* = 1.000), and between patients diagnosed with others and those who did not (*P* = 0.109) (Supplementary Fig. 2).

## Discussion

Fondaparinux is a factor Xa inhibitor, which has been applied to prevent VTE in various patients, including patients receiving orthopedic surgery or trauma surgery [14, 17, 21–24]. For example, a study conducted in Europe reports that fondaparinux (4%) exerts a better effect in preventing VTE occurrence compared to enoxaparin (9%) in patients receiving hip replacement surgery [25]. In addition, a study performed in America claims that fondaparinux (1.5%) effectively reduces the occurrence rate of VTE compared to dalteparin (2.1%), enoxaparin (2.3%), and unfractionated heparin (4.2%) in patients receiving orthopedic surgery; meanwhile, the occurrence rate of bleeding is lower in patients receiving fondaparinux (1.5%) compared to patients receiving unfractionated heparin (25%) [26]. Moreover, a previous study carried out in America reveals that the occurrence rate of VTE is lower in patients receiving fondaparinux (12.5%) compared to those receiving enoxaparin (27.8%) [17]. However, since fondaparinux is just released in China lately, relevant evidence regarding the effect of fondaparinux in Chinese patients is still limited. The current study discovered that the occurrence rate of in-hospital VTE, major bleeding, and death was 2.5%, 21.8%, and 0.0% in patients receiving orthopedic surgery or trauma surgery, which was in line with other studies (mentioned above) [25, 26]. It turned out that fondaparinux is effective in Chinese patients receiving orthopedic surgery or trauma surgery. The possible explanations would be that (1) fondaparinux could bind to antithrombin, leading to rapid inhibition of factor Xa, which affected the course of the coagulation cascade reaction, thereby suppressing thrombin formation and thrombus enlargement [27], and (2) fondaparinux formed a stable complex with PF4, which could enhance platelet aggregation and activation, thus resulting in a low incidence of major bleeding events [13].

Meanwhile, the current study also found that age > 60 years was a strong risk factor for the occurrence of in-hospital VTE in patients receiving fondaparinux after orthopedic surgery or trauma surgery. A possible explanation would be that older patients (age > 60 years) had higher hemoconcentration and therefore more likely to have in-hospital VTE. Apart from that, this study also discovered that osteoarthritis, femoral head necrosis, hip replacement, and knee replacement were independently linked to a higher risk of in-hospital major bleeding in patients receiving fondaparinux after orthopedic surgery or trauma surgery. The potential reason would be that osteoarthritis and femoral head necrosis could cause intraarticular bleeding, while hip replacement and knee replacement would directly damage the blood vessels near the bone, which further contributed to the major bleeding [28–30]. Besides, abnormal serum creatinine was also an independent risk factor for in-hospital major bleeding. It could be explained by that the abnormal serum creatinine represented dysfunction of the kidney, which contributed to the decrease in platelet counts [31]. Therefore, abnormal serum creatinine was correlated with increased in-hospital major bleeding risk.

Moreover, rare studies report adverse events in patients receiving fondaparinux after orthopedic surgery or trauma surgery [16, 32]. The current study discovered that the most common adverse events that occurred in patients receiving fondaparinux after orthopedic surgery or trauma surgery were surgical complications (59.6%) (including pain, wound bleeding, increased drainage, and infection), adverse reactions at injection site (0.4%), and other adverse events (11.0%). Notably, most of the adverse events were manageable and tolerable, indicating that fondaparinux had a good safety profile for patients receiving orthopedic surgery or trauma surgery.

Notably, several limitations should be noticed: (1) although 1258 patients were enrolled in this study, the administration of anticoagulants for orthopedic surgery or trauma surgery was quite prevalent and general. Therefore, the base of patients using anticoagulants would be large, indicating that the sample size could be further expanded; (2) this was not a comparative study; thus, a double-arm study was warranted to compare the efficacy of fondaparinux with other anticoagulants (such as unfractionated heparin, enoxaparin, and dabigatran) in patients receiving orthopedic surgery or trauma surgery; and (3) this study only explored the occurrence rate of in-hospital VTE, major bleeding, and death; thus, the long-term effect of fondaparinux in patients receiving orthopedic surgery or trauma surgery still needed exploration.

In summary, fondaparinux achieves low occurrence rates of in-hospital VTE and major bleeding with a good safety profile in patients receiving orthopedic surgery or trauma surgery. However, further validation is still required.

### Supplementary Information

Below is the link to the electronic supplementary material.

Supplementary file1 (TIF 1272 kb)

Supplementary file2 (TIF 2294 kb)

## Data Availability

The original contributions presented in the study are included in the article/supplementary material, further inquiries can be directed to the corresponding author.

## References

[1] Akhter S, Mundi R, Bhandari M (2020). The impact of evidence in surgery of the musculoskeletal system. World J Surg.

[2] Karachalios TS, Koutalos AA, Komnos GA (2020). Total hip arthroplasty in patients with osteoporosis. Hip Int.

[3] McClain WD, DeFoor MT, Patzkowski JC (2021). meniscus repair techniques. Sports Med Arthrosc Rev.

[4] Demirel M, Polat G, Ersen A (2021). Internal fixation for osteochondritis dissecans lesions of the knee in patients with physeal closure. Acta Orthop Traumatol Turc.

[5] Majima T, Oshima Y (2021). Venous thromboembolism in major orthopedic surgery. J Nippon Med Sch.

[6] Zambelli R, Nemeth B, Touw CE (2021). High risk of venous thromboembolism after orthopedic surgery in patients with thrombophilia. J Thromb Haemost.

[7] Khan F, Tritschler T, Kahn SR, Rodger MA (2021). Venous thromboembolism. Lancet.

[8] Falck-Ytter Y, Francis CW, Johanson NA (2012). Prevention of VTE in orthopedic surgery patients: antithrombotic therapy and prevention of thrombosis, 9th ed: American College of Chest Physicians Evidence-Based Clinical Practice Guidelines. Chest.

[9] Milling TJ, Ziebell CM (2020). A review of oral anticoagulants, old and new, in major bleeding and the need for urgent surgery. Trends Cardiovasc Med.

[10] Gomez-Outes A, Alcubilla P, Calvo-Rojas G (2021). Meta-analysis of reversal agents for severe bleeding associated with direct oral anticoagulants. J Am Coll Cardiol.

[11] Kleiboer B, Layer MA, Cafuir LA (2022). Postoperative bleeding complications in patients with hemophilia undergoing major orthopedic surgery: a prospective multicenter observational study. J Thromb Haemost.

[12] Zhang Y, Zhang M, Tan L (2019). The clinical use of fondaparinux: a synthetic heparin pentasaccharide. Prog Mol Biol Transl Sci.

[13] Chen LY, Khan N, Lindenbauer A, Nguyen TH (2022). When will fondaparinux induce thrombocytopenia?. Bioconjug Chem.

[14] Fu D, Li L, Li Y (2022). Fondaparinux sodium and low molecular weight heparin for venous thromboembolism prophylaxis in Chinese patients with major orthopedic surgery or trauma: a real-world study. BMC Surg.

[15] Singelyn FJ, Verheyen CC, Piovella F (2007). The safety and efficacy of extended thromboprophylaxis with fondaparinux after major orthopedic surgery of the lower limb with or without a neuraxial or deep peripheral nerve catheter: the EXPERT Study. Anesth Analg.

[16] Turpie AG, Bauer KA, Eriksson BI (2002). Postoperative fondaparinux versus postoperative enoxaparin for prevention of venous thromboembolism after elective hip-replacement surgery: a randomised double-blind trial. Lancet.

[17] Bauer KA, Eriksson BI, Lassen MR (2001). Fondaparinux compared with enoxaparin for the prevention of venous thromboembolism after elective major knee surgery. N Engl J Med.

[18] Eriksson BI, Bauer KA, Lassen MR (2001). Fondaparinux compared with enoxaparin for the prevention of venous thromboembolism after hip-fracture surgery. N Engl J Med.

[19] Kim KI, Kim GB, Lee MG, Song SJ (2021). Do we need chemoprophylaxis to prevent venous thromboembolism following medial open-wedge high tibial osteotomy?. J Knee Surg.

[20] Farge D, Frere C, Connors JM (2019). 2019 international clinical practice guidelines for the treatment and prophylaxis of venous thromboembolism in patients with cancer. Lancet Oncol.

[21] He T, Han F, Wang J (2021). Efficacy and safety of anticoagulants for postoperative thrombophylaxis in total hip and knee arthroplasty: a PRISMA-compliant Bayesian network meta-analysis. PLoS ONE.

[22] Kolluri R, Plessa AL, Sanders MC (2016). A randomized study of the safety and efficacy of fondaparinux versus placebo in the prevention of venous thromboembolism after coronary artery bypass graft surgery. Am Heart J.

[23] Tokuhara K, Matsushima H, Ueyama Y (2017). Efficacy and safety of thromboembolism prophylaxis with fondaparinux in Japanese colorectal cancer patients undergoing laparoscopic surgery: a phase II study. Int J Surg.

[24] Senzolo M, Piano S, Shalaby S (2021). Comparison of fondaparinux and low-molecular-weight heparin in the treatment of portal vein thrombosis in cirrhosis. Am J Med.

[25] Lassen MR, Bauer KA, Eriksson BI (2002). Postoperative fondaparinux versus preoperative enoxaparin for prevention of venous thromboembolism in elective hip-replacement surgery: a randomised double-blind comparison. Lancet.

[26] Shorr AF, Kwong LM, Sarnes M (2007). Venous thromboembolism after orthopedic surgery: implications of the choice for prophylaxis. Thromb Res.

[27] Zhou Z, Zhang L, Wu X (2022). Chemical synthesis and pharmacological properties of heparin pentasaccharide analogues. Eur J Med Chem.

[28] Lotz MK, Kraus VB (2010). New developments in osteoarthritis. Posttraumatic osteoarthritis: pathogenesis and pharmacological treatment options. Arthritis Res Ther.

[29] Beck M, Siebenrock KA, Affolter B (2004). Increased intraarticular pressure reduces blood flow to the femoral head. Clin Orthop Relat Res.

[30] Oberweis BS, Nukala S, Rosenberg A (2013). Thrombotic and bleeding complications after orthopedic surgery. Am Heart J.

[31] Boccardo P, Remuzzi G, Galbusera M (2004). Platelet dysfunction in renal failure. Semin Thromb Hemost.

[32] Tran AH, Lee G (2003). Fondaparinux for prevention of venous thromboembolism in major orthopedic surgery. Ann Pharmacother.

