# Supplementary figures and images for: Occurrence rates and risk factors of in-hospital venous thromboembolism, major bleeding, and death in patients receiving fondaparinux after orthopedic surgery or trauma surgery
Source: Ir J Med Sci. 2023 Feb 14;192(6):2973–9. doi: 10.1007/s11845-023-03289-7 (PMC10692026; doi:10.1007/s11845-023-03289-7)

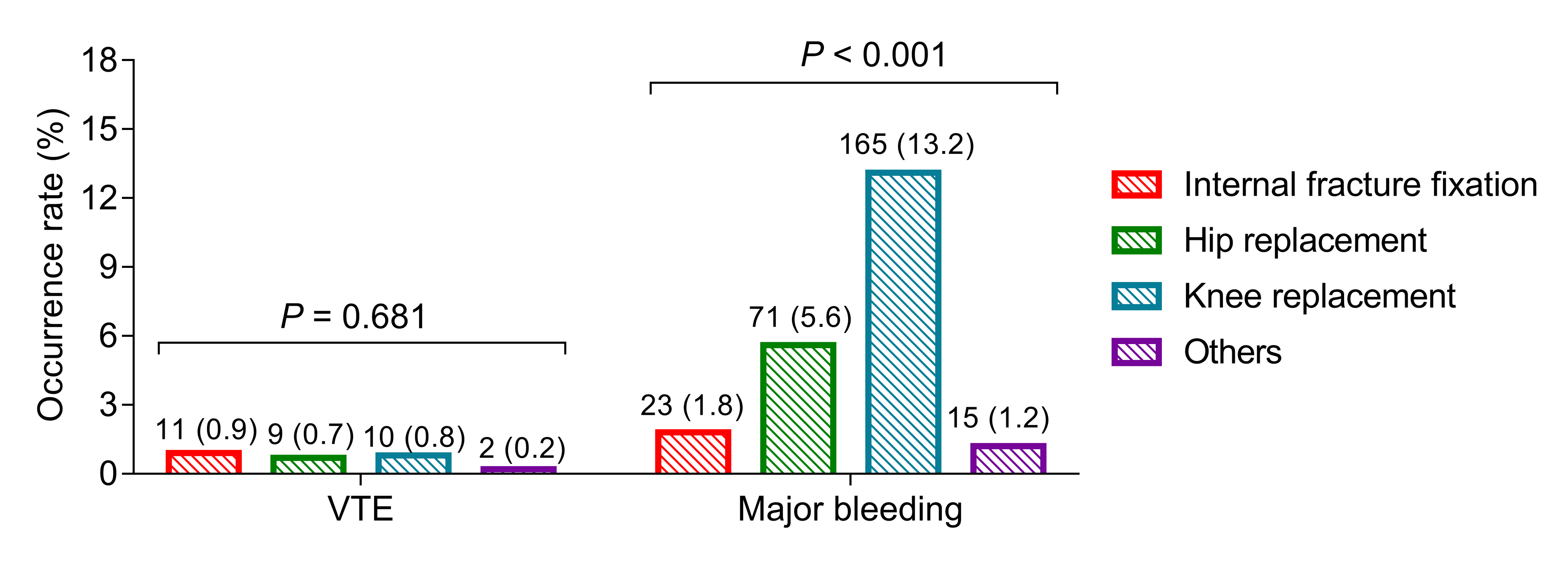

Supplement: Supplementary file 1 — Supplementary file1 (TIF 1272 kb) [file 11845_2023_3289_MOESM1_ESM.tif]

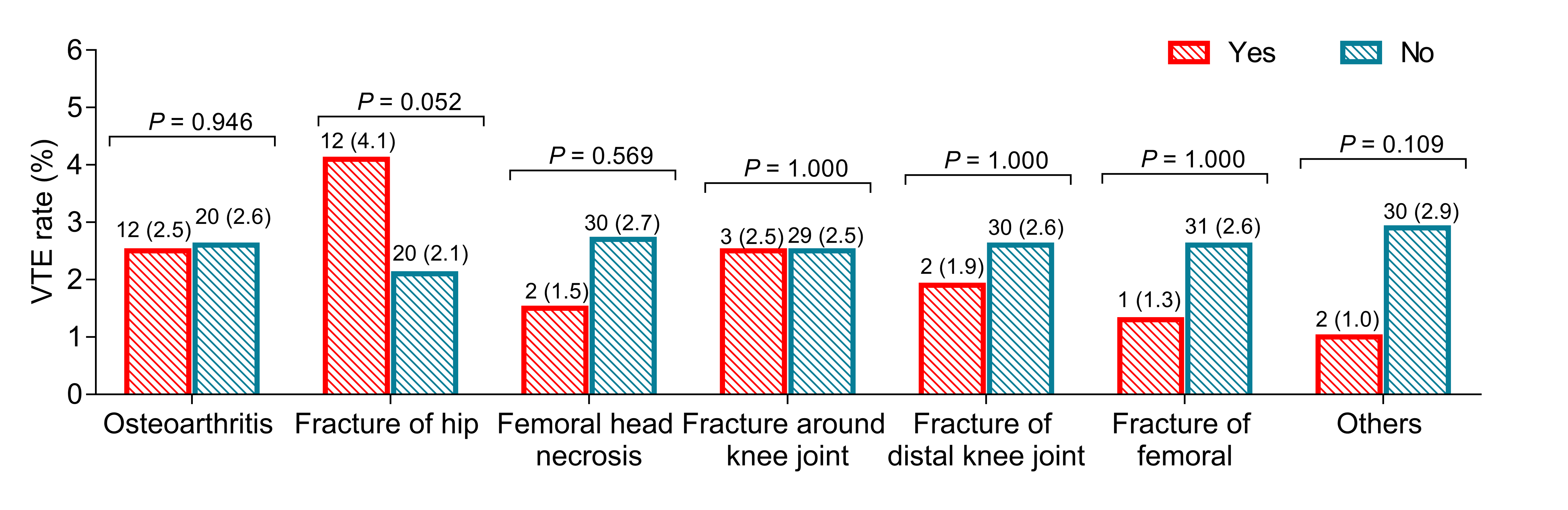

Supplement: Supplementary file 2 — Supplementary file2 (TIF 2294 kb) [file 11845_2023_3289_MOESM2_ESM.tif]
